# Nanomaterials-enabled point-of-care diagnostics for pathogens

**DOI:** 10.3389/fmed.2026.1931968

**Published:** 2026-08-27

**Authors:** Jiao Wu, Dengchao Wang, Wanping Ye, Juan He, Yayun Jiang

**Affiliations:** 1 Department of Clinical Laboratory, Mianzhu People's Hospital, Mianzhu City, Sichuan, China; 2 Department of Psychosomatic Medicine, Deyang People's Hospital, Deyang, Sichuan, China; 3 Department of Clinical Laboratory, Deyang People's Hospital, Deyang, Sichuan, China

**Keywords:** biosensors, nanomaterials, pathogen detection, POCT, signal amplification

## Abstract

Nanomaterials, with their unique physicochemical properties, have emerged as important enabling components for improving the performance of point-of-care testing (POCT) technologies for pathogen detection. Many conventional POCT methods still face challenges in sensitivity, specificity, multiplexing capability, and quantitative accuracy, particularly when applied to low-abundance pathogens in complex clinical samples. This review first summarizes the functional roles of representative nanomaterials in pathogen POCT, including optical signal output, catalytic signal amplification, target enrichment, and interfacial regulation. We then discuss how these materials improve lateral flow assays, biosensors, microfluidic systems, and isothermal nucleic acid amplification platforms. Finally, we summarize three major nanomaterial-assisted recognition mechanisms, including pathogen surface recognition, ROS-mediated signal conversion, and nucleic acid recognition, and discuss their applications in respiratory, urinary, and fecal specimens. These advances are expected to support the development of more sensitive, integrated, and field-deployable diagnostic systems for infectious disease control.

## 1 Introduction

The proliferation and dissemination of pathogens pose a significant threat to global public health and social development. Microbial infection remains a primary cause of morbidity and mortality worldwide (1). Pathogenic microorganisms have had a profound influence on human civilization throughout history, shaping its course. Examples include plague, cholera, and influenza pandemics (2). In the contemporary medical landscape, significant achievements have been made in the development of antimicrobial agents and vaccines. However, the challenges posed by pathogenic microorganisms are becoming increasingly severe and complex. The emergence and subsequent dissemination of antibiotic-resistant strains, a consequence of the excessive utilization of antibiotics, has once again engendered a scenario wherein the treatment of numerous prevalent bacterial infections is confronted with a paucity of efficacious pharmaceutical interventions (3). The process of global integration has also accelerated the speed and scope of transmission for emerging and re-emerging infectious diseases. The global pandemic of COVID-19 serves as the most salient example of this phenomenon, underscoring the vulnerability of global public health systems (4). Infections caused by pathogens continue to impose a significant economic burden on healthcare systems worldwide and have been demonstrated to increase patients' mortality and disability risks (5). Consequently, the development of rapid and precise pathogen detection technologies is imperative for effective prevention and control of infectious diseases. To address this need, a diverse array of detection methodologies has been developed for clinical and public health practice. These methods can be broadly categorized based on the biomarker they target: traditional culture-based techniques, immunoassays, and nucleic acid amplification tests. Each category offers distinct advantages and exhibits specific limitations in sensitivity, specificity, turnaround time, and applicability for identifying novel pathogens.

Traditional culture-based methods, which rely on pathogen growth, remain foundational because they enable the isolation of live pathogens and subsequent antimicrobial susceptibility testing. However, their utility is limited by prolonged turnaround times. Immunoassays, including techniques like immunochromatography and enzyme-linked immunosorbent assays (ELISAs), are based on specific antigen-antibody binding. These technologies offer advantages such as simple operation, rapid results, and low cost, making them suitable for primary healthcare facilities and rapid field screening (6). A related serological approach, host-based immune response testing, involves detecting specific antibodies in serum to evaluate infection history and immune status (7). Nucleic acid-based methods, such as real-time quantitative PCR, enable highly sensitive and specific identification by amplifying target gene sequences and are widely used as reference methods for detecting many pathogens (8). Furthermore, technological advances in metagenomic techniques enable direct high-throughput sequencing of all microbial nucleic acids in a sample, allowing for the identification of pathogens without prior knowledge (9). Despite their maturity, these conventional diagnostic platforms exhibit significant limitations, particularly in epidemic control and field deployment, as starkly highlighted by the COVID-19 pandemic. However, these methods still face limitations in point-of-care settings, including the prolonged turnaround time of culture-based methods, the variable sensitivity of some immunoassays, and the requirement for sophisticated instruments and trained personnel for molecular techniques.

In this context, point-of-care testing (POCT) has emerged as an important strategy for rapid pathogen diagnosis. POCT enables diagnostic procedures to be performed at or near the patient, thereby shortening turnaround time and supporting timely clinical decision-making (10). Typical POCT platforms include lateral flow immunoassay strips (11), microfluidic chips (12), and biosensors (13). However, many current POCT systems still face challenges in detecting low-abundance pathogens, achieving reliable multiplexing, and maintaining quantitative accuracy in complex clinical specimens (14). Therefore, innovative strategies are needed to enhance signal generation, improve target recognition, reduce matrix interference, and simplify detection workflows. Nanomaterials, with their high surface-to-volume ratio, tunable optical/electrical/magnetic properties, and facile surface functionalization, provide a promising route to address these challenges.

Recent advances in nanotechnology provide powerful tools for addressing these limitations. Owing to their size-dependent effects, large specific surface area, tunable optical/electrical/magnetic properties, and facile surface functionalization, nanomaterials have been widely incorporated into POCT platforms as signal labels, signal amplifiers, target-enrichment carriers, and sensing-interface modifiers (15–17). Their high loading capacity and programmable surfaces enable efficient immobilization of antibodies, aptamers, and nucleic acid probes, thereby improving target recognition and reducing matrix interference (18, 19). In addition, nanomaterials can generate diverse signal outputs, including colorimetric changes based on nanoparticle aggregation and fluorescence responses mediated by distance-dependent fluorescence resonance energy transfer (FRET) (20, 21). These features support the development of more sensitive, multiplexed, quantitative, and field-deployable pathogen POCT systems.

**Figure 1:**
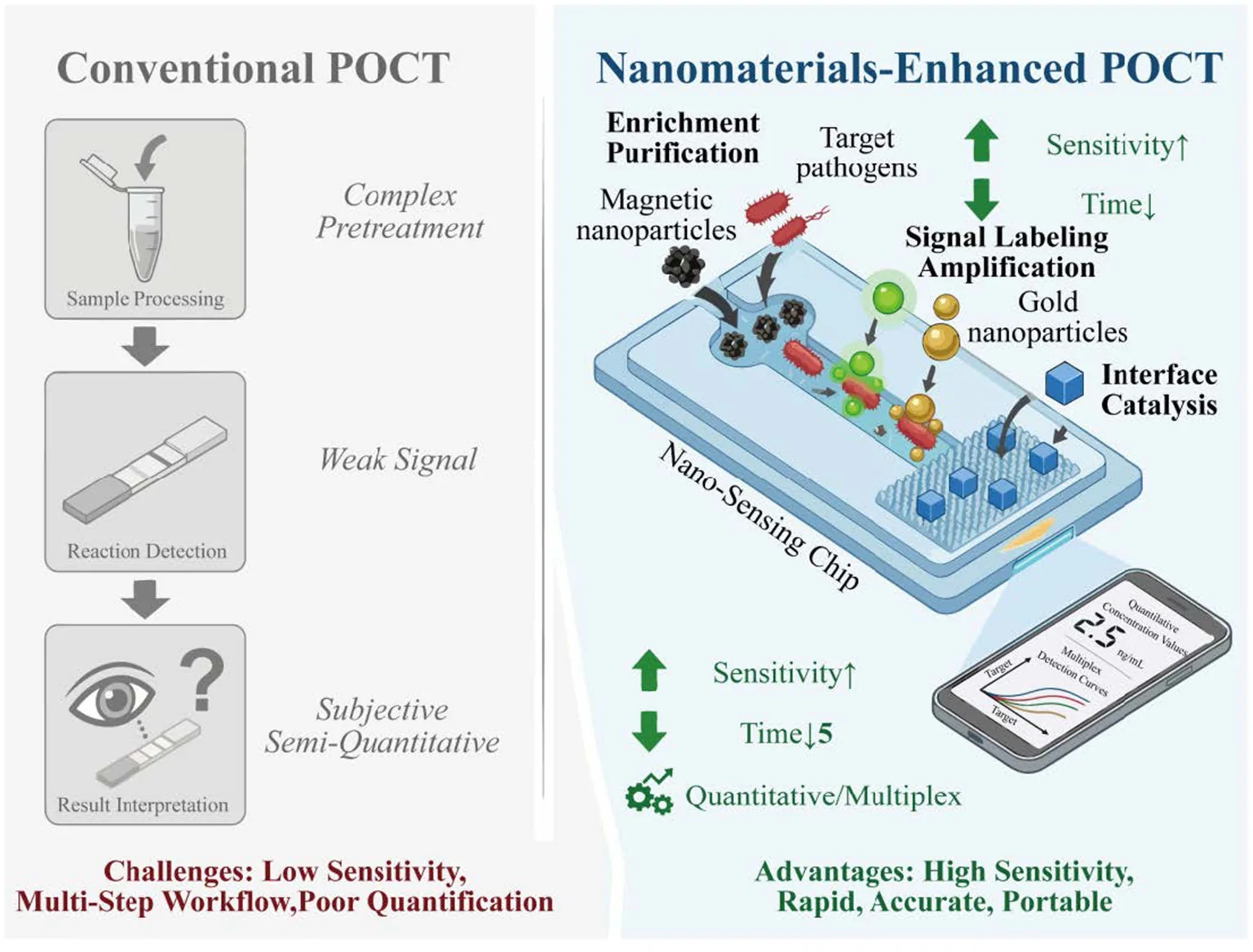
Conceptual comparison of conventional and nanomaterial-enhanced POCT workflows. Nanomaterials improve diagnostic performance by enabling target enrichment, signal amplification, and quantitative readout, thereby addressing key limitations of conventional platforms, including complex sample processing, limited signal output, and subjective result interpretation.

As illustrated in Figure 1, the integration of nanomaterials can substantially improve the analytical performance and operational workflow of POCT. Conventional POCT procedures (illustrated in Figure 1, left) frequently encounter challenges, including limited sensitivity, cumbersome steps, and pre-dominantly qualitative outcomes. In contrast, nanomaterial-enhanced POCT systems integrate functional components such as magnetic nanomaterials, plasmonic nanoparticles, fluorescent nanoprobes, and nanozymes to improve sample pre-treatment, target enrichment, signal amplification, and result interpretation. This progression highlights the transition of POCT from simple qualitative screening toward rapid, quantitative, and integrated diagnosis. This review comprehensively discusses the functional roles of nanomaterials in pathogen POCT, their integration into representative POCT platforms, the underlying recognition and signal-conversion mechanisms, specimen-specific applications, and the remaining challenges for clinical translation. This article presents a narrative review with a critical perspective on nanomaterial-enabled POCT technologies for pathogen detection. Relevant studies were identified through searches of PubMed, Web of Science, and Scopus using combinations of keywords, including “nanomaterials,” “nanoparticles,” “point-of-care testing,” “pathogen detection,” “biosensors,” “microfluidics,” “lateral flow assay,” “CRISPR,” and “isothermal amplification.” English-language literature published between 2015 and 2025 was primarily considered. Studies were selected based on their relevance to nanomaterial applications in pathogen POCT, technological advances, analytical performance, and clinical translation potential. Representative studies were prioritized to highlight key advances, underlying mechanisms, and remaining challenges in this field.

## 2 Functional roles of nanomaterials in pathogen POCT

**Figure 2:**
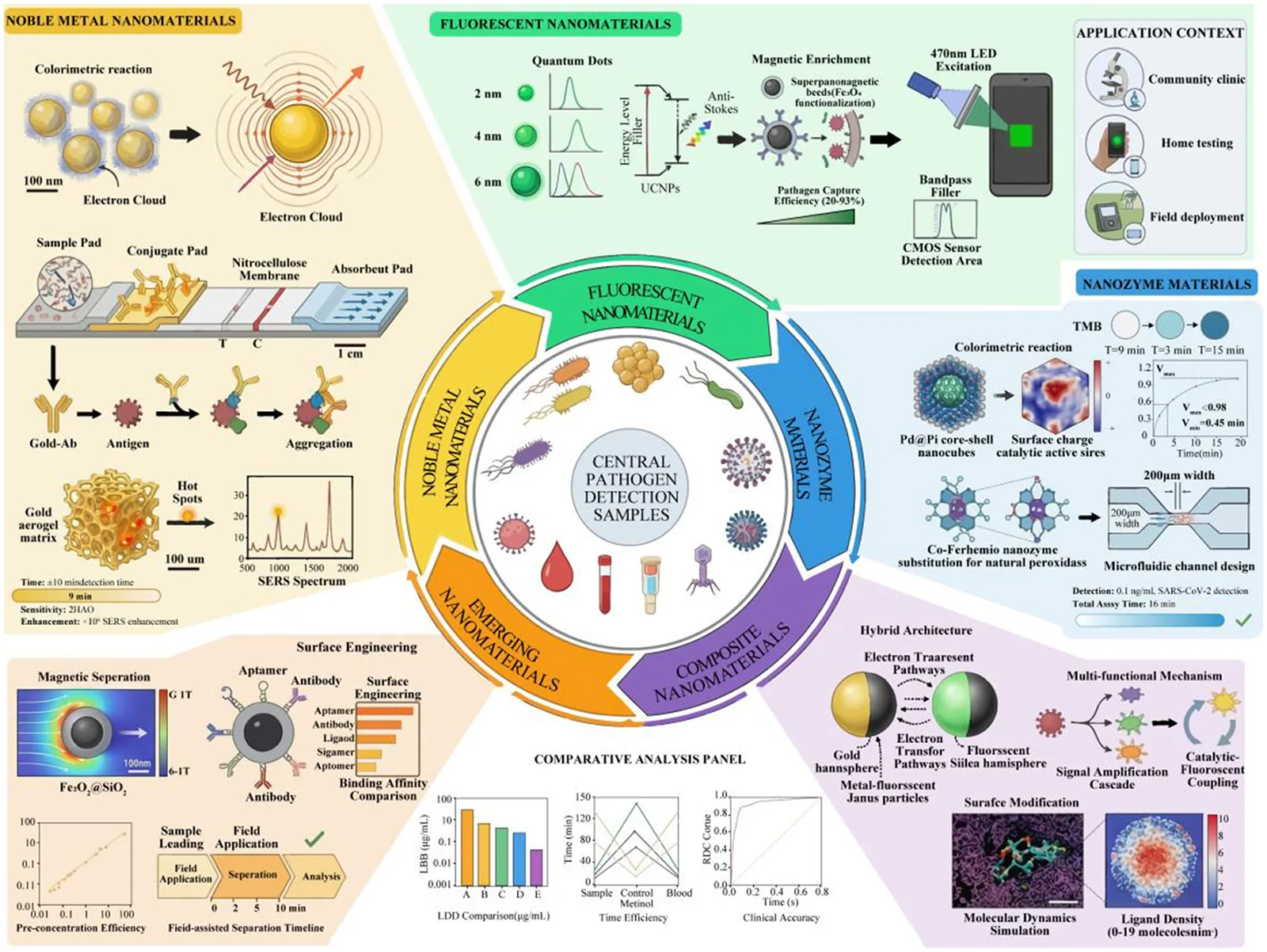
Classification and functional roles of representative nanomaterials in pathogen POCT. Various nanomaterials, including noble metal nanoparticles, fluorescent nanomaterials, magnetic nanomaterials, nanozymes, composite nanomaterials, and emerging nanomaterials, contribute to pathogen detection through optical signaling, target enrichment, catalytic amplification, and interface engineering.

Nanomaterials have become essential functional components for overcoming the inherent limitations of conventional POCT platforms. In pathogen POCT, representative nanomaterials can be discussed according to their pre-dominant roles in the detection process. Colorimetric and fluorescent nanomaterials mainly provide readable optical signals, magnetic nanomaterials facilitate target enrichment and sample purification, and catalytic nanomaterials such as nanozymes enable signal amplification. Although these categories are not mutually exclusive, they collectively illustrate how nanomaterials improve sensitivity, specificity, operational simplicity, and field applicability in pathogen POCT. As summarized in Figure 2, nanomaterials used in pathogen POCT can be broadly classified into several representative categories, including noble metal nanomaterials, fluorescent nanomaterials, magnetic nanomaterials, nanozyme-based materials, composite nanomaterials, and emerging nanomaterials. By exploiting their distinct optical, magnetic, catalytic, and interfacial properties, these nanomaterials provide versatile functions for signal generation, target enrichment, and assay performance enhancement in POCT systems.

Colorimetric nanomaterials, typically represented by noble metal nanoparticles (e.g., gold and silver), enable visual detection through the localized surface plasmon resonance (LSPR) effect (22). When incident light resonates with free electrons on the nanoparticle surface, it produces strong absorption in the visible spectrum, causing gold nanoparticles to exhibit stable and vivid colors. A prime example is the PHE-CoV immunochromatographic test strip developed by Keyan Chen et al. The test strip operates by preparing colloidal gold particles approximately 30 nm in diameter and labeling them with specific antibodies. When the target virus is present, the gold-labeled antibodies are captured and enriched at the test line. The intense Surface Plasmon Resonance (SPR) effect manifests as a vivid red band visible to the naked eye. This method completes detection within 10 min with a sensitivity of 2 hemagglutination units, demonstrating high consistency with RT-PCR results and showcasing the high sensitivity and operational simplicity of SPR-driven colorimetric detection (23). Thus, colorimetric nanomaterials have become core components for constructing user-friendly, instrument-free POCT platforms for pathogenic microorganisms.

Fluorescent nanomaterials, including quantum dots, upconversion nanoparticles, and dye-doped nanomaterials, have emerged as pivotal tools for enhancing POCT sensitivity and enabling simultaneous multi-target detection. This is primarily attributable to their inherent advantages, such as high quantum yield, photostability, and tunable emission wavelengths (24). Jiaxuan Li et al. developed dual-functional magnetic multilayer quantum dot nanocomposites (MagMQD@Si+), which combined bacterial enrichment with strong fluorescence output. This approach achieved ultra-low detection limits of 8–40 cells ml^−1^ across various bacterial strains and demonstrated rapid, specific point-of-care diagnostic capabilities in clinical sputum samples (25). Rui Yang et al. (26) coupled quantum dot fluorescent microspheres with the PRRSV envelope protein to prepare an immunochromatographic strip, which showed a detection limit of only 1.2 ng ml^−1^ for viral antibodies and a sensitivity of 96.7%, highlighting the efficiency of fluorescent nanomaterials in rapid serological virus screening. The dual-modal quantum dot/gold nanoparticle label developed by Tian Guan et al. (27) combines color visualization with inverse-ratio fluorescence dual readout, detecting bacterial antigen at 10 CFU ml^−1^ and demonstrating enhanced quantitative accuracy and signal-to-noise ratio. Through precise control of size, composition, and doping, multicolor fluorescence signals spanning the visible to near-infrared spectrum can be generated, enabling seamless interfacing with portable optical readers such as smartphones. This enables semi-quantitative or even fully quantitative POCT, significantly expanding its applicability in primary healthcare and resource-limited settings.

Magnetic nanomaterials, such as iron oxide (Fe_3_O_4_) nanoparticles and magnetic nanobeads, offer unique advantages for sample pre-treatment and target enrichment in complex matrices. By functionalizing magnetic nanoparticles with specific recognition elements (e.g., antibodies, aptamers, or antibiotics), target pathogens can be rapidly captured from large-volume samples and concentrated using an external magnetic field, thereby improving detection sensitivity and reducing matrix interference. For example, Jia-Jia Wang et al. (28) developed multifunctional cellular beacons with in-situ synthesized quantum dots, coupled with immunomagnetic separation, enabling naked-eye detection of enterovirus 71 via gold nanoparticle aggregation with a detection limit of 0.5 ng ml^−1^, and also allowing counting-based detection with a limit of 1 ng ml^−1^. Weiqiang Li et al. (29) employed vancomycin-functionalized magnetic beads (MBs-PEI-Van) combined with an MXene@Au-based electrochemical biosensor for the highly sensitive detection of methicillin-resistant *Staphylococcus aureus* in cerebrospinal fluid, achieving a detection limit of 3.8 × 10^1^ CFU ml^−1^. The integration of magnetic enrichment with downstream detection platforms (electrochemical, fluorescent, or colorimetric) provides a robust strategy for developing automated, sample-to-answer POCT systems.

Catalytic nanomaterials (nanozymes) combine the structural characteristics of nanomaterials with the catalytic functions of natural enzymes, including peroxidase-like, oxidase-like, and catalase-like activities. Compared to natural enzymes, nanozymes offer high chemical stability, low preparation cost, ease of large-scale production, and long-term storage capability (30). In pathogen POCT, nanozymes catalyze substrate reactions to generate colorimetric, luminescent, or electrochemical signals, enabling continuous signal amplification and significantly lowering detection limits. Unlike conventional colorimetric nanomaterials that mainly serve as optical labels, catalytic nanomaterials improve detection performance primarily through enzyme-like signal amplification. Cheng et al. (31) developed a chromatographic immunoassay using Pd@Pt nanozyme-based dual-antibody technology to detect *Salmonella Enteritidis* and *Escherichia coli* O157:H7, with detection limits of 20 and 34 CFU ml^−1^, respectively, and results directly readable by smartphone. Liu et al. (32) developed a Co-Fe/heme nanozyme-based chemiluminescent paper strip that replaced HRP with a Co-Fe/heme complex nanozyme, achieving rapid and ultrasensitive detection of the SARS-CoV-2 S protein. Leng et al. (33) reported a Hemin@MI nanozyme-based colorimetric immunosensor that generated a visually detectable blue signal through TMB conversion, with a detection limit of 8.5 × 105 CFU ml^−1^ for *E. coli* O157:H7 on-site screening. By leveraging their efficient catalytic properties and signal-amplification capabilities, nanozymes enable rapid and sensitive detection of low-abundance pathogens on immunochromatographic, chemiluminescent, or colorimetric platforms, providing robust technical support for rapid pathogen detection in resource-limited and point-of-care settings.

## 3 Integration of nanomaterials into major POCT platforms

Beyond their intrinsic functional properties, nanomaterials can be integrated into different POCT platforms to address platform-specific limitations. In lateral flow assays, they mainly improve visual signal intensity, quantitative readout, and multiplex detection. In biosensors, they enhance electron transfer, optical response, target capture, and interfacial reaction efficiency. In microfluidic systems, they facilitate sample pre-treatment, enrichment, and automated signal generation. In isothermal amplification platforms, they convert nucleic acid amplification products into readable optical or electrochemical signals. In lateral flow immunoassays (LFIAs), nanomaterials markedly enhance detection sensitivity and signal diversification through multiple mechanisms. With regard to enhancing visibility, noble metal nanoparticles have been shown to optimize color intensity via the LSPR effect. For instance, silver nanoparticles developed by Lufsyi Mahmudin et al. (34) reduced the detection limit for *E. coli* to 0.47 CFU ml^−1^. Zhihao Li et al. (35) further reduced the detection limit to 10 CFU ml^−1^ using a surface-enhanced Raman scattering (SERS) multimodal system constructed with Au@Pt core-shell nanoenzymes. Additionally, the elevated specific surface area and surface programmability of nanomaterials facilitate multi-target detection. In a significant development, Xin Zhang's research team pioneered a novel approach by integrating functionalized graphene quantum dots with machine learning algorithms, enabling the concurrent identification of five distinct pathogen types (with a detection limit of 10^3^ CFU ml^−1^) (36). In summary, nanomaterials have been shown to enhance LFIA performance by facilitating the transition from qualitative to quantitative analysis and enabling multi-target analysis. Additionally, they have been shown to systematically improve the overall performance of POCT platforms through multi-level signal amplification and precise interface regulation.

Biosensors offer a highly adaptable technical approach for pathogen POCT by converting biological recognition events into measurable physical or chemical signals. The incorporation of nanomaterials into biosensors primarily results in systematic enhancements in signal transduction efficiency, interfacial reaction kinetics, and target enrichment capabilities (37). Electrochemical biosensors have gradually become an important component in the POCT field due to their advantages of rapid detection, high degree of miniaturization, and ease of quantitative analysis. For instance, Y. Veera Manohara Reddy et al. significantly enhanced the specific surface area and electron transfer efficiency of electrodes by modifying them with NiO-rGO/MXene nanocomposites. This amplification of electrical signals, triggered by pathogen binding, enabled highly sensitive detection of influenza viruses (e.g., H1N1 and H5N2) with a detection limit as low as 3.63 nanomoles (38). The incorporation of magnetic nanoparticles has emerged as a highly effective approach for the selective enrichment of target pathogens in complex biological specimens. Cong-Ying Wen et al. developed a new method for the rapid capture of bacteria (e.g., *Salmonella*) at ultra-low concentrations using magnetic nanospheres (MNs). This method utilizes an external magnetic field to achieve capture efficiencies of up to 97%, and it can even detect single bacteria (39). The integration of this magnetic enrichment strategy with electrochemical detection techniques has been shown to further enhance the detection performance. Richa Pandey et al. employed DNAzyme-immobilized microgel magnetic beads to achieve rapid, specific, culture-free, and wash-free electrochemical quantification of bacteria in untreated urine, with a detection limit as low as 6 CFU ml^−1^. This result demonstrates the high robustness of the “magnetic enrichment-electrochemical detection” model (40). In the domain of optical sensing, photobiological sensors demonstrate remarkable sensitivity and multiplex detection potential when enhanced by nanomaterials. In their study, Chongwen Wang et al. utilized a combination of vancomycin-modified Ag-coated magnetic nanoparticles and secondary-enhanced Au@Ag nanoparticles to expedite bacterial detection through SERS. The detection limit was determined to be 5 × 10^2^ cells ml^−1^, with excellent specificity (41). Concurrently, substantial advancements have been made in nanoscale sensing systems based on FRET: Nuo Duan et al. developed an aptamer-based biosensor that utilizes dual FRET effects from quantum dots to carbon nanoparticles for the simultaneous detection of multiple pathogens (e.g., *Vibrio parahaemolyticus* and *Salmonella*). This biosensor achieves detection limits as low as 25–35 CFU ml^−1^. This underscores the efficacy of optical sensors in multi-target parallel detection (42). The utilization of optical sensors is of paramount importance in the construction of high-performance POCT platforms, as they provide essential technical support. The enhancement of signal transduction efficiency, interfacial enrichment capabilities, and optical/electrochemical amplification effects by nanomaterials has enabled biosensors to achieve substantial advances in analytical performance, transitioning from qualitative detection to highly sensitive, semi-quantitative, and even fully quantitative analysis in POCT. The formidable challenge of matrix interference in complex samples, a key limitation of POCT noted earlier, demands biosensors with exceptional stability. A case in point is the detection of biomarkers in urine. A key requirement for POCT biosensors is maintaining stability in harsh matrices. A case in point is the detection of biomarkers in urine, where high ionic strength and pH fluctuations can destabilize sensor interfaces. Yangkun Feng et al. (43) addressed this by developing an iodine-doped perovskite nanocatalyst (CsPbI_3_) with exceptional chemical stability and catalytic efficiency, thereby enabling robust colorimetric detection of NMP22 in raw urine samples. This highlights how engineered nanomaterials can confer the necessary interference resistance to biosensors for real-world applications.

**Figure 3:**
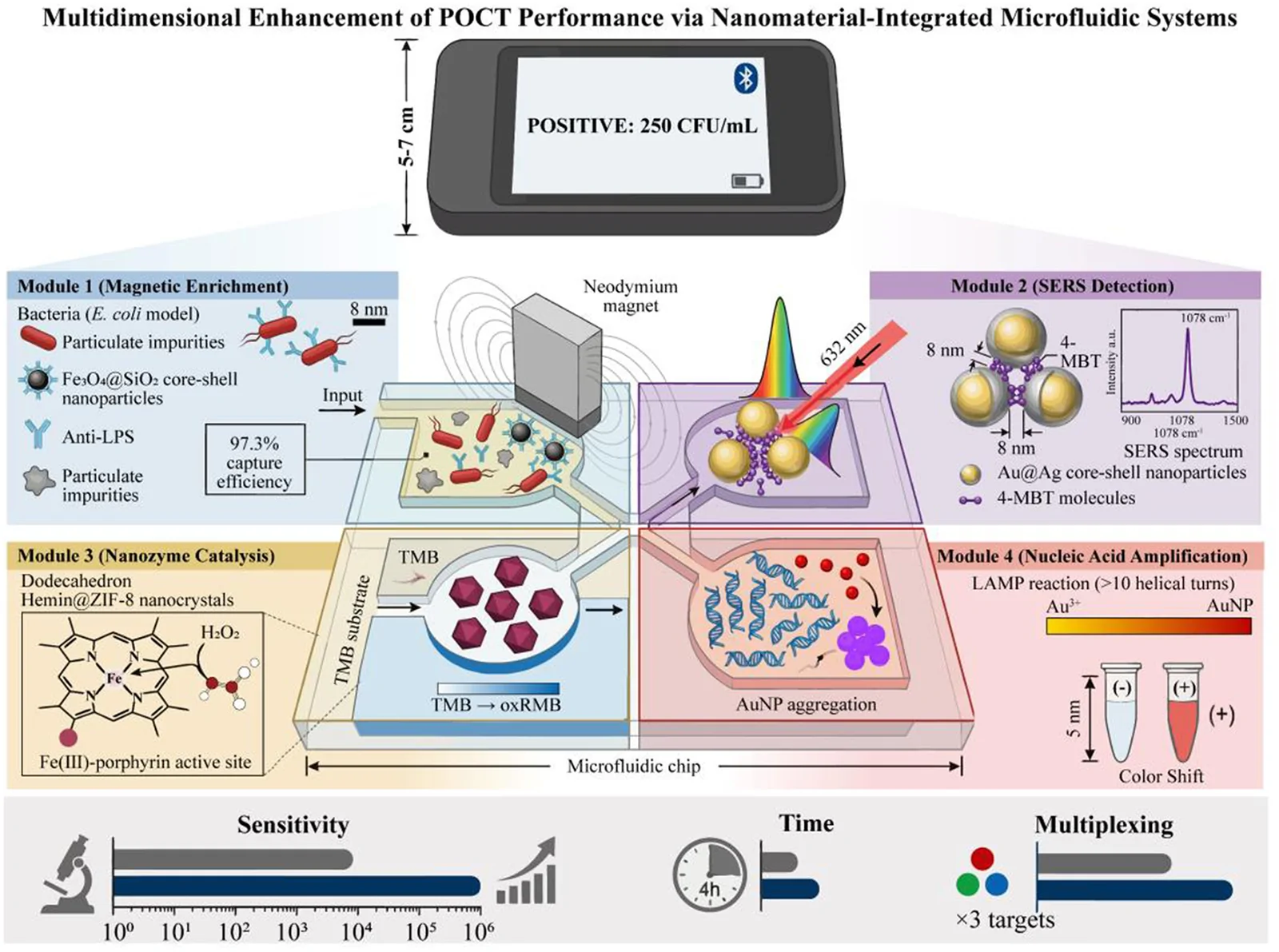
Conceptual design of a nanomaterial-integrated microfluidic platform for enhanced pathogen POCT performance. The integration of magnetic enrichment, SERS detection, nanozyme catalysis, and nucleic acid amplification modules enables improved target capture, signal amplification, and multiplex detection within a compact diagnostic workflow.

Microfluidic technology facilitates the precise manipulation of minute liquid volumes at the chip scale, thereby providing a highly integrated and automated reaction platform for POCT (44). As illustrated in Figure 3, the integration of nanomaterials with microfluidic systems enables an integrated diagnostic workflow by combining sample preparation, pathogen enrichment, signal amplification, and detection within a compact platform, providing a promising strategy for sample-in and result-out POCT. The integration of nanomaterials is particularly crucial for processing complex clinical samples. For instance, the analysis of fecal samples, which contain intricate microbial compositions and inhibitory substances, poses a significant challenge. The challenge of analyzing complex samples like feces is effectively addressed by integrating nanomaterials with microfluidics. For instance, Shanshan Feng et al. developed a microfluidic platform integrated with aptamer-functionalized gold-coated magnetic nanoparticles (Au@Fe_3_O_4_ NPs) (45). This system enabled the efficient capture and enrichment of *Parvimonas* from the complex fecal matrix, followed by a nanozyme-based colorimetric detection, achieving a low detection limit. This example demonstrates how the “microfluidics-nanomaterials” combination effectively manages sample complexity for point-of-care gut pathogen detection. However, reliance on microfluidic structures alone is often inadequate for detecting low-abundance pathogens. The integration of nanomaterials and microfluidic technology is identified as a pivotal approach for developing integrated POCT systems, which are designed to facilitate the “sample-in, result-out” functionality (46). Such integrated platforms incorporate multiple nanomaterial-enhanced functional modules into modular microfluidic chips, enabling coordinated sample preparation, pathogen enrichment, signal amplification, and result interpretation within a unified workflow. Microfluidic structures control fluid transport and reaction timing, whereas nanomaterials provide target capture and signal amplification. This combination reduces manual operation and improves the feasibility of sample-in–result-out POCT.

For example, magnetic nanoparticles enable rapid capture and enrichment of pathogens within chips. Vancomycin-functionalized magnetic beads (Van-MBs), developed by Yutong Wang et al., employ antibiotic-mediated magnetic separation technology combined with rolling circle amplification (RCA) to generate abundant single-stranded DNA probes that emit fluorescent signals. This strategy enabled highly sensitive detection of *S. aureus* in fruit juice, with a detection limit of 3.3 × 10^2^ CFU ml^−1^ (47). In the domain of signal detection, a study by Chongwen Wang et al. employed vancomycin-modified silver-coated magnetic nanoparticles (Fe_3_O_4_@Ag-Van MNPs) for pathogen enrichment. The use of Au@Ag core-shell nanoparticles as secondary enhancers to construct SERS “hotspots” has been shown to facilitate the detection of multiple bacteria (e.g., Escherichia coli and methicillin-resistant *S. aureus*) within 30 min, with a detection limit as low as 5 × 10^2^ cells ml^−1^ (41). Through the rational design of microchannel structures and the distribution of nanomaterials, highly integrated multi-step detection processes can be achieved on a single chip, thereby significantly reducing detection time and minimizing human operational errors. Jiashu Sun et al. (48) have indicated that integrating gold nanoparticles with microfluidic technology has the potential to advance POCT devices toward portability and automation through multimodal sensing strategies, including colorimetry, electrochemistry, SPR, and SERS. This “microfluidics-nanomaterials” synergistic strategy, through nano-mediated target enrichment and signal amplification mechanisms, provides crucial technical support for the development of fully automated, portable POCT platforms for pathogenic microorganisms.

The reliance of conventional PCR on temperature-controlled equipment and specific operating conditions severely limits its application in POCT scenarios. Isothermal nucleic acid amplification technologies, such as Loop-mediated Isothermal Amplification (LAMP), offer a viable solution for point-of-care testing by enabling nucleic acid amplification under constant temperature conditions (49). The employment of nanomaterials in this domain has led to significant advancements in the sensitivity, readability, and operational efficiency of isothermal amplification systems (50). Specifically, nanomaterials have been shown to serve as signal-amplification tags, thereby converting minute amplification into highly sensitive electrochemical or optical signals. In the domain of electrochemical sensing, the aptasensor developed by Huasong Bai et al. employs cocoon-like DNA nanostructures (CDNs). The synthesis of these nanostructures is achieved through RCA, which enables the incorporation of repetitive G-quadruplex units. The presence of hemin results in the formation of DNA enzymes that exhibit peroxidase-like activity. These enzymes facilitate the reduction of H_2_O_2_, thereby generating electrochemical signals. This sensor demonstrated high sensitivity and specificity for detecting *E. coli* O157:H7, with a detection limit of 10 CFU ml^−1^. This feature eliminates the necessity for complex nucleic acid extraction steps, which are time-consuming and laborious. This underscores the efficacy of nanomaterials in enhancing detection sensitivity (51). In the field of optical signal conversion, nanomaterials have been shown to generate multimodal outputs owing to their distinctive optical properties. For instance, the strategy of ultraviolet-induced *in situ* gold nanoparticle formation proposed by Rajamanickam Sivakumar's team combines LAMP amplification with colorimetric detection: it has been established that nitrogenous bases in amplification products can capture gold ions. This process leads to the formation of gold nanoparticles upon UV irradiation and sodium citrate reduction. This color change indicates a shift in the solution's state from colorless to red. This change enables the solution to be detected visually. This method can detect the presence of SARS-CoV-2 plasmid DNA in a sample within 30 min, with a detection limit of 42 fg μl^−1^ (52). The integration of nanomaterials with technologies such as LAMP facilitates rapid, highly sensitive detection of pathogen nucleic acids without complex instrumentation, making it particularly suitable for primary healthcare settings and resource-constrained environments.

Overall, nanomaterials have substantially improved signal transduction and amplification strategies in POCT systems by enhancing target enrichment, signal generation, and readout sensitivity. This has enabled traditional POCT technologies to achieve a qualitative leap in sensitivity, specificity, and functional integration. To facilitate technology selection for different clinical applications, we provide a comparative analysis of representative nanomaterial platform combinations based on key performance metrics. In lateral flow immunoassays, noble metal nanoparticles enable instrument-free visual readout but are generally limited to qualitative or semi-quantitative detection. Fluorescent nanomaterials can improve sensitivity by 10 - 100-fold (LOD: 10^1^−10^3^ CFU ml^−1^) but require portable fluorescence readers. Nanozymes provide an intermediate strategy by introducing catalytic amplification (LOD: 10^0^−10^2^ CFU ml^−1^) while retaining visual interpretation, although additional substrate incubation steps may be required (31, 32). In biosensor platforms, electrochemical systems offer quantitative analysis and miniaturization potential (LOD: 10^0^−10^2^ CFU ml^−1^), whereas optical sensors such as SERS provide excellent sensitivity and multiplexing capability but often require more sophisticated instrumentation (38, 41). Microfluidic systems enable integrated sample processing and sample-in–result-out workflows with reduced sample consumption. However, their broader application remains limited by higher fabrication costs and scalability challenges compared with strip-based systems (48). These comparative characteristics are summarized in Table 1. These advances expand the application scope of pathogen POCT and provide opportunities for developing next-generation diagnostic platforms.

**Table 1:** Comparative performance analysis of nanomaterial-platform combinations.

Nanomaterial type	Platform	Target pathogen/specimen	LOD	Assay time	Key advantages	Key limitations	References
Gold nanoparticles	LFAs (colorimetric)	PHE-CoV (respiratory)	2 HA units	10 min	Visual readout, simple, low cost	Qualitative only, limited sensitivity	(23)
Quantum dots	LFIA (fluorescent)	Multiple bacteria (sputum)	8–40 cells ml^−1^	~15 min	High sensitivity, multiplexing capability	Requires fluorescence reader	(25)
Pd@Pt Nanozyme	LFIA (catalytic)	*Salmonella*/*E. coli* (food)	20–34 CFU ml^−1^	15–20 min	Signal amplification, smartphone readout	Substrate addition step	(31)
NiO-rGO/MXene	Electrochemical biosensor	Influenza virus (clinical)	3.63 nm	20–30 min	Quantitative, miniaturizable	Requires trained operator	(38)
Ag-coated MNPs	SERS biosensor	*S. aureus*/MRSA (culture)	5 × 10^2^ cells ml^−1^	30 min	High specificity, multiplex potential	Complex instrumentation	(41)
Au@Fe_3_O_4_ NPs	Microfluidic + colorimetric	*P. micra* (feces)	11 CFU ml^−1^	~60 min	Sample prep integrated, aptamer-based	Complex chip fabrication	(45)
Iron oxide MNPs	IMA + LFIA	Influenza A (saliva)	3 × 10^3^−4 × 104 PFU ml^−1^	< 30 min	Matrix interference reduction, enrichment	Requires magnetic separation	(53)
CsPbI_3_ Perovskite	Colorimetric nanozyme	NMP22 (urine)	0.03 U ml^−1^	~20 min	Stable in urine, high catalytic efficiency	Indirect biomarker detection	(43)
Gold Nanoparticles	CRISPR/Cas12a-LFD	ASFV (blood)	~2 μg (gene)	73 min	High specificity, nucleic acid detection	Multi-step workflow	(54)
Au@Pt Nanozymes	Catalytic-SERS LFIA	Pathogens (milk)	10 CFU ml^−1^	20 min	Dual-modal (catalytic + SERS)	Complex probe synthesis	(35)

LFIA, lateral flow immunoassay; LOD, limit of detection; PHE-CoV, porcine hemagglutinating encephalomyelitis virus; HA, hemagglutination; MRSA, methicillin-resistant S. aureus; SERS, surface-enhanced Raman spectroscopy; MNPs, magnetic nanoparticles; IMA, immunomagnetic assay; NMP22, nuclear matrix protein 22; ASFV, African swine fever virus; CFU, colony-forming units; PFU, plaque-forming units.

## 4 Recognition and signal-conversion mechanisms enhanced by nanomaterials

POCT for pathogenic microorganisms is predicated on the efficient identification and precise conversion of their characteristic biological information. Compared with conventional laboratory testing, POCT emphasizes rapid, reliable, and user-friendly detection of analytes in complex sample settings. Consequently, the mechanisms responsible for its detection must exhibit high specificity, incorporate signal amplification strategies, and be highly compatible with portable testing platforms. In nanomaterial-enabled pathogen POCT systems, detection generally involves two interconnected processes: target recognition and signal conversion. Target recognition is mainly achieved through pathogen surface recognition or nucleic acid recognition, whereas signal conversion is often enhanced by nanozyme-mediated catalytic reactions, including ROS-related reactions (55–57). Among these, detection mechanisms based on membrane surface interactions are among the most intuitive and mature strategies in point-of-care testing for pathogenic microorganisms.

The surfaces of pathogens, including bacteria and viruses, exhibit highly conserved molecular structures that are of biological significance. These structures include lipopolysaccharides (LPS) in bacterial cell walls (58), lipopeptidoglycan (59), peptidoglycan (60), and viral envelope or capsid proteins (61). As demonstrated by the experiments, the electrochemical immunosensor targeting LPS antibodies enables rapid and specific detection at low concentrations as low as 2 CFU ml^−1^. This validates the feasibility of LPS as a conserved marker on bacterial surfaces (62). Similarly, polysulfide aptamer sensors that were truncated and targeted the SARS-CoV-2 spike protein achieved an ultra-low detection limit of 8 fg ml^−1^ in milligram-scale samples, with no cross-reactivity to other viral proteins. This further demonstrates that the conservation of viral envelope proteins can be exploited for highly sensitive POCT (63). Flexible, metal-based PET immunosensors have been shown to detect sub-picomolar levels of the S protein in synthetic saliva. The results obtained are in full agreement with those achieved by conventional ELISA assays (64). The collective findings of these studies lend support to the core proposition that surface-conserved molecular structures serve as ideal targets for pathogen POCT, providing robust experimental evidence for the rapid diagnosis of microbial systems enabled by nanomaterials. However, several specificity challenges should be considered. For surface-based recognition, LPS heterogeneity among Gram-negative bacteria, particularly the diversity of O-antigen polysaccharides, may result in antibody cross-reactivity with non-target pathogens or reduced detection of variant strains. Nanomaterial-based strategies may partially mitigate these limitations through aptamer cocktails or machine learning-enabled sensor arrays that differentiate pathogens based on binding patterns (65); however, these approaches do not fully resolve the underlying biological variability. Similarly, spike protein variation among SARS-CoV-2 variants may reduce the performance of antibody-based immunoassays. Although aptamers targeting conserved epitopes may provide improved adaptability, their development increases assay complexity. For nucleic acid-based detection, pathogen genome evolution may introduce mutations within primer or probe binding regions, potentially causing false-negative results. CRISPR/Cas systems targeting multiple conserved genomic regions can reduce this risk; however, continuous genomic surveillance and periodic assay updates remain necessary (66). Cross-reactivity with closely related pathogens further emphasizes the need for careful target selection, sequence design, and comprehensive specificity evaluation. Furthermore, nanomaterials alone cannot fully overcome these biological challenges. Although high-density functionalization can enhance avidity, it may also increase nonspecific interactions. In addition, heterogeneous surface modification may contribute to batch-to-batch variation, while complex sample matrices can compromise specificity in real-world applications (67). Achieving reliable specificity requires integrated strategies, including multiplex detection, machine learning-assisted interpretation, sample pre-treatment, and multi-epitope recognition. Despite these advances, specificity remains a major analytical challenge in clinical POCT, and prospective clinical validation studies are essential to determine performance in intended-use populations.

The specific capture of pathogenic microorganisms can be achieved by modifying the surfaces of nanomaterials with antibodies, nucleic acid aptamers, functional peptides, or small-molecule ligands. Junfeng Wang et al. employed the hydroxylamine seeding method to expedite the formation of rough gold shells. The research team successfully achieved magnetic capture and surface-enhanced Raman scattering detection of *S. aureus* LPS by conjugating polyethyleneimine (PEI)-modified gold-coated magnetic nanoparticles (AuMNPs) with antibodies. The AuMNPs were formed via the hydroxylamine seeding method, which involves the rapid formation of a rough gold shell. These were then coupled with *S. aureus* antibodies to form capture probes. The amplification of signals was accomplished through the utilization of an AuNR-DTNB SERS tag. The results demonstrated a detection limit of 10 cells per milliliter and linearity over the range of 10^1^−10^5^ cells per milliliter. This achieved approximately 10-fold signal amplification (68). In the study by Yeun-Chung Chang et al. (69) *S. aureus*-targeting nucleic acid aptamers were covalently attached to the surface of gold nanoparticles to construct an ‘aptamer-AuNP' detection system. The findings of these studies demonstrate the efficient capture and signal transduction capabilities of nucleic acid aptamer-functionalized nanomaterials for POCT. Ghadeer A. R. Y. Suaifan et al. designed magnetic nanoparticles (MNPs) containing a specific peptide substrate that can be cleaved by proteases produced by *Staphylococcus aureus*. Following cleavage, the MNPs disengage from the black surface, exposing the gold substrate. A discernible color shift occurred within 1 min, thereby enabling rapid, instrument-independent detection (70).

This strategy demonstrates that, following surface modification of nanomaterials, small-molecule peptide ligands can directly convert capture events into readable signals via interfacial color changes. When target pathogens bind to these functionalized nanoprobes, aggregation, conformational changes, or alterations to the interfacial properties of the nanomaterials are triggered. These physical changes are then converted into color shifts, fluorescent signals, or electrochemical responses, enabling rapid detection.

In recent years, research into detection mechanisms based on ROS regulation or response has become increasingly popular. For instance, in the mercaptophenylboronic acid-mediated nanozyme immunochromatography platform, the multi-arm magnetic gold/iridium nanozyme catalyzes the generation of abundant ROS upon pathogen capture. Subsequent to capture, the nanozyme catalyzes the production of hydroxyl radicals (·OH) from H_2_O_2_/AEC substrates on the test line. These then oxidize AEC, forming an insoluble red precipitate (oxAEC) and thereby achieving signal amplification. The post-catalytic detection limit is remarkably low, at 1.2 pg/ml for the SARS-CoV-2 spike protein and 17/21 cells ml^−1^ for Pseudomonas aeruginosa and Streptococcus pneumoniae, respectively. This enhancement results in an overall sensitivity that is 238–416-fold greater than that of conventional colloidal gold immunochromatography, thereby expanding the detection range by over two orders of magnitude (71). Mucus-mimicking, ciliated nanozyme hydrogel composites utilize glucose oxidase, which is released from lysed bacteria, to drive H_2_O_2_ production and ROS generation. Subsequently, the FePDA nanozymes convert H_2_O_2_ into •OH radicals, which in turn trigger a colorimetric reaction that achieves rapid detection at the level of 10 CFU ml^−1^ (72). Nanozyme-based, multi-array, gas-driven sensors catalyze H_2_O_2_ decomposition to generate O_2_, and the resulting gas pressure is converted into visual displacement, enabling ultrasensitive on-site quantification of Group A Streptococcus (73). These studies indicate that nanozyme-mediated catalytic systems can convert pathogen-triggered catalytic reactions into colorimetric, fluorescent, electrochemical, or pressure-based signals. This methodology facilitates indirect assessment of microbial activity, viability, and even drug susceptibility. This mechanism offers a novel approach to dynamic monitoring of pathogenic microorganisms, making it suitable for POCT applications, such as evaluating antimicrobial treatment efficacy and rapid drug-susceptibility screening.

Nucleic acid recognition-based detection mechanisms are considered a primary strategy for achieving high sensitivity and specificity. A study conducted by Xinjie Wang et al. has yielded findings that suggest the development of a detection platform (CRISPR/Cas12a-LFD) that integrates CRISPR/Cas12a technology with a gold-labeled immunochromatographic strip. This platform has the potential for portable detection of the African swine fever virus (ASFV). This platform utilizes the Cas12a-crRNA complex to specifically recognize ASFV genomic DNA and activate transcleavage activity. It also employs gold nanoparticle-labeled immunochromatographic strips for signal conversion and amplification. Specifically, the system employs a gold nanoparticle-labeled mouse anti-digoxigenin antibody as the signal probe. Upon Cas12a cleavage of the digoxigenin/biotin dual-labeled ssDNA reporter molecule, the free digoxigenin moiety is captured by antibodies on the test line of the strip, leading to gold nanoparticle enrichment and the formation of a visible detection line (54). Liang Xiaoyan et al. combined recombinase polymerase amplification (RPA) with CRISPR-Cas12a to achieve signal amplification on a lateral flow strip using gold nanoparticles labeled with streptavidin-tagged reporter probes. This method achieved a detection limit of only 2 μg for the FUM1 gene, with the entire process completed in 73 min. It facilitates on-site DNA extraction, RPA, Cas12a cleavage, and interpretation of results, yielding outcomes consistent with traditional single-spore isolation sequencing (74). The immobilization of complementary nucleic acid probes, CRISPR-associated recognition elements, or nucleic acid aptamers onto nanomaterial surfaces facilitates high-density probe loading and signal amplification. This method rapidly and accurately converts pathogen nucleic acid information into colorimetric, fluorescent, or electrochemical responses, thereby establishing a robust technical foundation for isothermal nucleic acid amplification technology and genotyping POCT detection.

## 5 Nanomaterial-enabled POCT in different clinical specimens

The distribution characteristics, background complexity, and inhibitory factors of pathogenic microorganisms vary significantly across different clinical samples. This variation imposes distinct demands on the sensitivity, interference resistance, and sample adaptability of POCT platforms (75). In respiratory samples such as nasopharyngeal swabs, saliva, and sputum, viral loads are typically low, and the samples are rich in interfering components like mucins, host nucleic acids, and proteins. These components can easily lead to nonspecific adsorption and signal suppression. To address this issue, Carole Farre and colleagues developed a biotin-coated silica nanoparticle-enhanced immunomagnetic assay, achieving highly sensitive detection of influenza A virus. The study utilized 50-nm silica nanoparticles with surface modifications consisting of short DNA spacer arms and an approximate loading of 50 biotin molecules. Subsequent to magnetic bead capture of viral nucleoproteins, biotin-labeled nanoparticles exhibited efficient amplification of the colorimetric signal through biotin-streptavidin-HRP conjugation. In simulated clinical samples containing 10% saliva, the detection limits for H1N1 and H3N2 viruses reached 3 × 10^3^ and 4 × 10^4^ PFU ml^−1^, respectively (53). This represents a 65 to 75-fold increase in sensitivity compared to traditional ELISA. This strategy reduces matrix interference through magnetic enrichment and amplifies the signal via the high specific surface area of nanoparticles, providing an effective technical pathway for rapid screening of respiratory viruses such as influenza and SARS-CoV-2. Furthermore, the low cost of nanomaterials like silica nanoparticles enhances the cost-effectiveness of such assays, making them suitable for large-scale screening in resource-limited settings. In clinical workflows, such technologies are particularly suitable for rapid influenza and SARS-CoV-2 screening in primary care settings, emergency departments, and community testing centers. For example, biotin-coated silica nanoparticle-enhanced immunomagnetic assays have demonstrated the potential to provide results within 30 min, supporting same-visit diagnosis and timely clinical decision-making regarding isolation or antiviral therapy. Similarly, nanozyme-based immunochromatographic assays may support the differential diagnosis of community-acquired pneumonia by improving detection performance and potentially assisting antibiotic stewardship.

Urine samples have become a pivotal target for POCT of urinary tract infections due to their non-invasive collection and ease of handling. It is important to distinguish between two major categories of urine-based diagnostics: direct pathogen detection, which identifies microorganisms or pathogen-derived components in urine, and host biomarker detection, which measures human proteins or metabolites associated with infection or disease. Although several representative urine-based POCT technologies reviewed in this article measure host biomarkers rather than directly identifying pathogens, they may offer novel approaches for urinary pathogen detection. Direct pathogen detection in urine presents unique challenges. Pathogen concentrations are often low (approximately 10^3^−10^5^ CFU ml^−1^ for significant bacteriuria), and the complex urine matrix contains high ionic strength, variable pH, and potential bacterial growth inhibitors that may interfere with detection. These matrix-related factors, particularly high ionic strength and pH variation, can compromise the stability of biosensor interfaces and analytical performance. To address this, nanocatalysts with excellent chemical stability and catalytic activity are expected to have far-reaching prospects. These catalysts utilize signal amplification mechanisms to convert weak target signals into stable, readable outputs. For instance, in catalytic colorimetric reactions, the iodine-doped perovskite nanocatalyst CsPbI3, developed by Yangkun Feng et al., exhibits a 24-fold enhancement in catalytic efficiency compared with conventional materials. It facilitates the detection of nuclear matrix protein 22 (NMP22) in urine with a detection limit of 0.03 U ml^−1^, exhibiting remarkable interference resistance in complex matrices (43). The economical synthesis of such nanocatalysts also reduces overall testing costs, promoting their adoption in routine POCT for urine samples.

The conversion of low-concentration target signals into stable, readable outputs is a pre-requisite for achieving rapid detection of urinary system-related biomarkers. Despite the limitations of direct nanocatalyst sensing for urinary pathogens, this technology has been successfully validated for detecting multiple targets in urine samples. For instance, the detection of glucose in urine (76), disease biomarkers (77), and drug molecules is possible (78). The integration of specific recognition elements with highly efficient nanocatalyst-reporting systems, along with performance optimization through strategies such as three-dimensional carriers and spatial confinement, collectively delineates a technologically coherent pathway. This approach provides a solid foundation for the future development of POCT methods targeting urinary tract pathogens. In clinical practice, urine-based technologies offer opportunities for non-invasive diagnosis across several clinical scenarios. For urinary tract infection screening, magnetic enrichment and nanozyme-based amplification strategies demonstrated for other targets may be adapted for rapid pathogen detection in primary care settings, potentially supporting same-visit clinical decision-making. Nanomaterial-based approaches for direct urinary pathogen detection may employ several design strategies, including magnetic enrichment using pathogen-specific recognition elements, nanozyme-mediated catalytic detection, CRISPR/Cas-based nucleic acid detection with improved sample preparation, and nanoparticle aggregation-based sensing. However, none of the reviewed technologies has yet achieved clinically validated direct urinary pathogen detection that meets routine diagnostic requirements, highlighting an important research gap and future priority.

Fecal samples present a more challenging matrix for detecting intestinal pathogens than urine. Their intricate microbial composition and copious inhibitory substances considerably impede the efficacy of conventional POCT technologies (79). In this scenario, the primary function of nanomaterials is their highly selective and efficient enrichment capabilities targeting specific pathogens or their nucleic acids. The development of nanoprobes with multivalent recognition capabilities has enabled the selective capture and isolation of specific pathogens, even in complex backgrounds. The use of functionalized nanoprobes enables high-sensitivity detection of low-abundance pathogens in feces. This is achieved through a combination of aptamer-mediated specific recognition and nanozyme signal amplification. For instance, Shanshan Feng et al. employed whole-bacterial SELEX to identify nucleic acid aptamers with high affinity (Kd = 33.84 nm) for *Parvimonas micra*, a bacterium associated with colorectal cancer. These aptamers were modified onto the surface of gold-coated ferrite nanoparticles (Au@Fe_3_O_4_ NPs) to construct capture probes. This probe has been shown to inhibit the nanomaterial's peroxidase-like activity by binding to the bacterium via the aptamer, thereby blocking the TMB colorimetric reaction. This method facilitates the quantitative detection of *P. micra*, exhibiting a linear range of 10^0^-10^8^ CFU ml^−1^ and a detection limit of 11 CFU ml^−1^. The cost-efficient production of gold-coated nanoparticles used in this probe underscores the affordability of nanomaterial-based POCT for intestinal pathogen detection in low-resource environments. In a validation using 18 clinical fecal samples, the sensor successfully distinguished colorectal cancer patients from healthy individuals (*p* < 0.05), with colorimetric signals highly consistent with those of quantitative polymerase chain reaction (qPCR) (45). Integrating this strategy with a microfluidic platform has the potential to achieve full process integration from sample pre-treatment to signal output, offering a practical solution for rapid screening of intestinal infections in primary healthcare settings. In clinical workflows, fecal-based nanotechnology platforms may facilitate rapid detection of enteric pathogens, particularly for foodborne pathogen surveillance and hospital-associated infection control. Magnetic bead-based enrichment systems may accelerate pathogen recovery and reduce reliance on lengthy culture-based confirmation procedures. In hospital settings, rapid fecal pathogen detection may support earlier infection control responses for *C. difficile*. However, the complexity of fecal matrix processing remains a major challenge, and these technologies may initially be more suitable for centralized laboratories rather than true point-of-care applications.

The structural characteristics, biological behavior, and infection mechanisms of different pathogenic microorganisms dictate the need for differentiated POCT detection strategies. The use of multifaceted functionalities, including surface functionalization, magnetic enrichment, signal amplification (electromagnetic hotspots, nanoenzymes, SPR), and multiplex coding, enables nanomaterials to serve as detection platforms for viruses, bacteria, and fungi. These platforms exhibit enhanced sensitivity, interference resistance, and expedited readout capabilities. This finding underscores the considerable advantages of nanomaterials in point-of-care diagnostics, antimicrobial susceptibility testing, and simultaneous detection of multiple pathogens. This provides robust technical support for the evolution of POCT from a “general-purpose tool” to a “precision diagnostic platform.” The diverse applications of nanomaterial-enabled POCT across different clinical specimens are summarized in Table 2, which compares representative platforms based on specimen type, target pathogens, analytical challenges, nanomaterial-based solutions, clinical applications, and key performance characteristics. For clarity, the key abbreviations used throughout this review are summarized in Table 3.

**Table 2:** Nanomaterial-enabled POCT by specimen type and clinical application.

Specimen type	Representative pathogens	Key challenges	Nanomaterial solutions	Clinical use case	Performance metrics	References
Respiratory (nasopharyngeal swab, saliva, sputum)	SARS-CoV-2, influenza A/B, RSV, S. pneumoniae	Low viral load, mucin interference, RNA degradation	Magnetic enrichment, silica NP signal amplification, nanozyme immunochromatography	Rapid triage in primary care, emergency departments; differential diagnosis of respiratory infections	LOD: 10^3^-104 PFU ml^−1^; Assay time: 10–30 min; Sensitivity: >95% vs. PCR	(32, 53, 71)
Urine	*E. coli, K. pneumoniae, P. aeruginosa* (UTIs); host biomarkers (NMP22, albumin)	High ionic strength, pH fluctuations, low pathogen concentrations	Perovskite nanozymes (stability), CNT-based electrodes (interference resistance), magnetic enrichment	Non-invasive UTI screening; CKD risk assessment; bladder cancer triage	LOD: 0.03 U ml^−1^ (NMP22); 60 nm (albumin); Assay time: 15–20 min	(43, 76, 80)
Fecal	*Salmonella, Campylobacter, C. difficile, P. micra* (colorectal cancer biomarker)	Complex matrix, PCR inhibitors, high background microbiota	Aptamer-functionalized magnetic NPs, microfluidic integration, TMB colorimetric detection	CRC risk screening; foodborne pathogen surveillance; outpatient intestinal infection diagnosis	LOD: 11–10^2^ CFU ml^−1^; Assay time: 30–60 min; Distinguishes CRC patients vs. healthy (*p* < 0.05)	(45, 81)
Blood/serum	HIV, HBV, HCV, sepsis-causing bacteria	Low pathogen concentration, high protein interference, hemolysis	Immunomagnetic separation, quantum dot fluorescent probes, CRISPR-Cas12a amplification	HIV/HBV/HCV screening in resource-limited settings; sepsis rapid diagnosis	LOD: 0.5 ng ml^−1^ (EV71); 1.2 pg/ml (S protein); Assay time: 30–73 min	(82, 83)

**Table 3:** . Glossary of key abbreviations used in this review.

Abbreviation	Full name	Abbreviation	Full name
POCT	Point-of-care testing	AuNPs	Gold nanoparticles
LFIA	Lateral flow immunoassay	AgNPs	Silver nanoparticles
LFA	Lateral flow assay	QDs	Quantum dots
LFD	Lateral flow detection	crRNA	CRISPR RNA
LAMP	Loop-mediated isothermal amplification	UCNPs	Upconversion nanoparticles
RPA	Recombinase polymerase amplification	DLS	Dynamic light scattering
RCA	Rolling circle amplification	TEM	Transmission electron microscopy
PCR	Polymerase chain reaction	UV–vis	Ultraviolet–visible spectroscopy
Cas	CRISPR-associated protein	HIV	Human immunodeficiency virus
MOFs	Metal–organic frameworks	HBV	Hepatitis B virus
FRET	Fluorescence resonance energy transfer	HCV	Hepatitis C virus
SERS	Surface-enhanced Raman scattering	ASFV	African swine fever virus
SPR	Surface plasmon resonance	LOD	Limit of detection
LSPR	Localized surface plasmon resonance	HA	Hemagglutination
ROS	Reactive oxygen species	SARS-CoV-2	Severe acute respiratory syndrome coronavirus 2
TMB	3,3′,5,5′-Tetramethylbenzidine	HRP	Horseradish peroxidase

## 6 Key challenges and future development trends in technology transfer

Despite substantial progress in laboratory research on nanomaterial-enabled POCT technologies, their large-scale clinical translation continues to face significant challenges (84). First, standardized nanomaterial preparation and batch-to-batch consistency remain major challenges for industrialization (85). Minor variations in size distribution, surface charge, and functionalization efficiency between synthesis batches may lead to fluctuations in diagnostic performance. Key quality control parameters include particle size and distribution (measured by DLS and TEM), surface charge (zeta potential), functionalization efficiency, aggregation state (UV–vis spectroscopy), and catalytic activity (standardized substrate conversion assays) (86). Current regulatory frameworks, including those of the FDA, EMA, and NMPA, lack fully harmonized guidelines specifically addressing nanodiagnostic products (87), and manufacturers often rely on internal reference materials for batch validation.

Biosafety assessment represents another critical requirement for clinical translation (88, 89). Although most POCT tests are designed for *in vitro* applications, the long-term safety implications of nanomaterials during sample processing, waste disposal, and environmental exposure require careful evaluation. Potential concerns include occupational exposure during manufacturing, disposal of nanomaterial-containing diagnostic waste, and possible environmental impacts on aquatic organisms. OECD guidelines for nanomaterial testing provide useful evaluation frameworks; however, comprehensive safety studies may be costly and time-consuming.

Third, regulatory pathways for nanomaterial-enabled diagnostic products remain evolving. Regulatory clearance pathways for nanomaterial-based POCT devices remain limited, and available routes generally depend on device characteristics and intended use. Major regulatory challenges include demonstrating clinical performance through multicenter studies, establishing stability specifications through accelerated aging and real-time stability testing, ensuring batch-to-batch consistency during manufacturing, and complying with quality management requirements such as ISO 13485 (90).

Cost and scalability are also critical determinants of real-world adoption. Although nanomaterials can improve assay sensitivity and signal generation (91), some platforms still require complex synthesis procedures, specialized surface modification, expensive readout devices, or controlled storage conditions. These limitations may restrict their implementation in primary care institutions and resource-limited settings (92). Future development should prioritize low-cost materials, scalable manufacturing processes, simplified workflows, room-temperature-stable reagents, and portable or instrument-free readout systems. For low- and middle-income settings, accessibility and equity should be considered as essential factors during assay design. Technologies requiring minimal equipment, limited technical training, and short assay times are more likely to support decentralized infectious disease diagnosis, community screening, and public health surveillance (93).

In the future, nanomaterial-enabled POCT technologies may evolve toward more integrated, intelligent, and accessible diagnostic systems (67). The combination of nanomaterials with microfluidics, flexible electronics, smartphone-based readers, and artificial intelligence-assisted analysis may enhance automation, data interpretation, and connectivity with digital health systems. However, successful translation will depend on addressing unresolved challenges related to manufacturing standardization, biosafety evaluation, regulatory pathways, cost control, scalability, and real-world clinical validation.

## 7 Conclusion

The integration of nanomaterials has substantially advanced POCT technologies by improving key aspects of the diagnostic process, including signal amplification, target enrichment, and multiplex detection. These advances have improved analytical sensitivity, signal generation, and operational efficiency in pathogen detection, whereas specificity remains largely dependent on the selection and performance of recognition elements. However, many reported performance metrics are derived from proof-of-concept studies conducted under optimized laboratory conditions, and multicenter clinical validation remains limited for most systems reviewed here.

Nanomaterials serve as versatile functional components that address specific performance bottlenecks in existing POCT platforms rather than replace established diagnostic approaches. The integration of nanotechnology with microfluidics, isothermal amplification, CRISPR-based detection, and artificial intelligence provides opportunities for developing more integrated diagnostic systems. These advances demonstrate that nanomaterials contribute to incremental but important improvements in diagnostic capabilities. Nevertheless, translating these integrated platforms from laboratory prototypes into clinically validated products requires addressing persistent challenges, including manufacturing standardization, regulatory pathways, cost-effectiveness, and scalable production. Future research should also address unresolved issues related to long-term biosafety evaluation, batch-to-batch variability, large-scale multicenter validation, and integration with digital health infrastructures, including electronic health records and AI-assisted decision support systems.

If these translational barriers can be addressed, nanomaterial-enabled POCT platforms may improve infectious disease diagnostics, particularly in resource-limited settings where conventional laboratory infrastructure is limited. Realizing this potential will require sustained efforts in clinical validation, manufacturing scale-up, regulatory harmonization, and equitable implementation. The continued evolution of nanotechnology, combined with advances in microfluidics and data science, may facilitate the gradual development of more sensitive, integrated, and accessible diagnostic solutions for global health needs through iterative technological improvements rather than revolutionary disruption.
